# Phytohormone balance and stress-related cellular responses are involved in the transition from bud to shoot growth in leafy spurge

**DOI:** 10.1186/s12870-016-0735-2

**Published:** 2016-02-20

**Authors:** Wun S. Chao, Münevver Doğramaci, David P. Horvath, James V. Anderson, Michael E. Foley

**Affiliations:** USDA-Agricultural Research Service, Biosciences Research Laboratory, 1605 Albrecht Boulevard, Fargo, ND 58102-2765 USA

**Keywords:** Dormancy, Hormone profiling, Leafy spurge, RNA-seq, Vegetative growth

## Abstract

**Background:**

Leafy spurge (*Euphorbia esula* L.) is an herbaceous weed that maintains a perennial growth pattern through seasonal production of abundant underground adventitious buds (UABs) on the crown and lateral roots. During the normal growing season, differentiation of bud to shoot growth is inhibited by physiological factors external to the affected structure; a phenomenon referred to as paradormancy. Initiation of shoot growth from paradormant UABs can be accomplished through removal of the aerial shoots (hereafter referred to as paradormancy release).

**Results:**

In this study, phytohormone abundance and the transcriptomes of paradormant UABs vs. shoot-induced growth at 6, 24, and 72 h after paradormancy release were compared based on hormone profiling and RNA-seq analyses. Results indicated that auxin, abscisic acid (ABA), and flavonoid signaling were involved in maintaining paradormancy in UABs of leafy spurge. However, auxin, ABA, and flavonoid levels/signals decreased by 6 h after paradormancy release, in conjunction with increase in gibberellic acid (GA), cytokinin, jasmonic acid (JA), ethylene, and brassinosteroid (BR) levels/signals. Twenty four h after paradormancy release, auxin and ABA levels/signals increased, in conjunction with increase in GA levels/signals. Major cellular changes were also identified in UABs at 24 h, since both principal component and Venn diagram analysis of transcriptomes clearly set the 24 h shoot-induced growth apart from other time groups. In addition, increase in auxin and ABA levels/signals and the down-regulation of 40 over-represented AraCyc pathways indicated that stress-derived cellular responses may be involved in the activation of stress-induced re-orientation required for initiation of shoot growth. Seventy two h after paradormancy release, auxin, cytokinin, and GA levels/signals were increased, whereas ABA, JA, and ethylene levels/signals were decreased.

**Conclusion:**

Combined results were consistent with different phytohormone signals acting in concert to direct cellular changes involved in bud differentiation and shoot growth. In addition, shifts in balance of these phytohormones at different time points and stress-related cellular responses after paradormancy release appear to be critical factors driving transition of bud to shoot growth.

**Electronic supplementary material:**

The online version of this article (doi:10.1186/s12870-016-0735-2) contains supplementary material, which is available to authorized users.

## Background

Leafy spurge (*Euphorbia esula* L.) is an herbaceous perennial weed that causes major economic losses in the Upper Great Plains of the United States [1, 2]. It maintains its perennial growth cycle through the seasonal production of abundant underground adventitious buds (UABs) on the crown and lateral roots (often referred to as crown and root buds). Dormancy in these UABs inhibits initiation of new vegetative growth under favorable or unfavorable environmental conditions and is an important survival mechanism [3]. Leafy spurge UABs are capable of manifesting the three well-defined phases of para-, endo-, and eco-dormancy [4]. Paradormancy is growth cessation controlled by physiological factors external to the affected structure, endodormacy is growth cessation controlled by internal physiological factors, and ecodormancy is growth cessation controlled by external environmental factors [5].

Signals originating from environmental and physiological factors during plant development are involved in facilitating the different phases of dormancy [6, 7]. Environmental signals such as temperature and light play crucial roles in regulating induction and release of bud dormancy, though the extent of their effects and the crosstalk between temperature- and light-regulated signaling pathways appear to be species dependent [7]. Physiological signals, including phytochrome, sugar, and phytohormones, are basically associated with direct phenotypic changes when plants perceive environmental signals.

Phytohormones that have been associated with bud growth and development include abscisic acid (ABA), ethylene, gibberellic acid (GA), cytokinin, brassinosteroids (BR), and auxin. ABA is involved in stress responses, bud development, and bud maturation [8–10] and may contribute to the suppression of growth during bud formation [9] and the development of endodormancy [11, 12]. Ethylene facilitates short day photoperiod-induced terminal bud formation, as well as normal endodormancy development [13, 14]. Ethylene is also required for ABA accumulation [13, 15] and may interact with ABA and auxin signaling pathways for apical dominance [14]. GA alone or in combination with other hormones regulates many aspects of plant growth and development [16] including vegetative bud growth (cell elongation) following dormancy release [6]. Cytokinins control cell division, shoot meristem initiation, leaf and root differentiation, and various aspects of plant growth and development [17]. Cytokinins also function as key regulatory signals promoting axillary bud outgrowth when the apical meristem is removed [18]. BRs are a class of naturally-occurring steroid phytohormones regulating essential physiological processes during plant growth and development. BR signaling interacts with light, GA and auxin pathways to regulate different aspects of photomorphogenesis [19, 20].

Auxin regulates numerous plant developmental and physiological processes [21], and auxin signaling has been well studied in paradormant buds. In general, auxin is synthesized in the primary shoot apex, moves basipetally through the stem, and inhibits axillary bud outgrowth [22]. Basipetal movement of auxin in the stem also affects the acropetal movement of cytokinin and strigolactone (secondary messengers), which promotes and inhibits bud outgrowth, respectively [23–26]. It is thought that the involvement of ABA on strigolactone biosynthesis could contribute to regulation of paradormancy in vegetative buds [27]. In addition, auxin-regulated strigolactone depletion is a major cause of branching after removal of the growing shoot apices [28]. Paradormancy in leafy spurge inhibits UABs from developing into new shoots through auxin and sugar signals generated from the actively growing aerial portion of the plant [29–32].

Leafy spurge has been used as a model perennial to investigate well-defined phases of dormancy in UABs [4, 33–36]. Further, development of an EST database [37] provided opportunities to study the transcriptome of leafy spurge UABs following paradormancy release [38]. Early results, obtained using a 2654-element Euphorbiaceae cDNA microarray, identified several differentially-regulated genes. For example, genes encoding putative homologues of asparagine synthase, a phosphate-inducible protein, and a curculin-like (mannose binding) lectin family protein were rapidly up-regulated and genes involved in flavonoid biosynthesis were rapidly down-regulated upon loss of paradormancy. To further investigate the regulation of gene expression during paradormancy release and initiation of shoot growth from crown buds following aerial stem removal, we compared the transcriptome of paradormant and growth-induced UABs based on RNA-seq data.

**Fig. 1:**
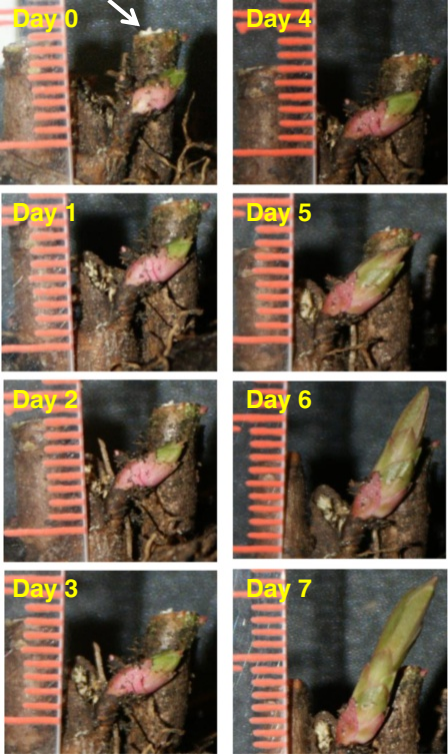
Growth of a crown bud after shoot removal. The arrow (Day 0) indicates where the shoot was excised

In this research, crown buds were harvested from paradormant leafy spurge plants (0 h) and also from plants post-decapitation of all aerial tissues (6, 24, and 72 h). Daily growth of a crown bud after shoot removal is shown in Fig. 1. These UABs were also used for hormone measurements and preparation of RNA samples for RNA-seq and RT-qPCR analyses. Based on the analyses of RNA-seq, RT-qPCR, and hormone profiling data, our results were consistent with different phytohormone signals acting in concert to direct cellular changes involved in growth; in addition, shifts in balance among these phytohormones at different time points and stress-related cellular responses after paradormancy release appear to be critical factors driving transition of bud to shoot growth.

## Results

### Principal component analysis indicates 24 h as the most active period of cellular changes during paradormancy release

**Fig. 2:**
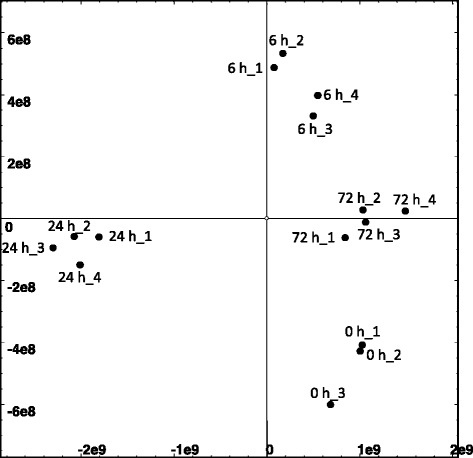
Principal component analysis applied to 7855 differentially-regulated genes (PPDE ≥ 0.95) based on RNA-seq analyses of underground adventitious buds at 0, 6, 24, and 72 h after released from paradormancy by shoot removal

RNA-seq technology was used to identify signaling pathways and differences in transcript profiles in leafy spurge crown buds during the transition from paradormancy to shoot-induced growth. Of the 569,227 contigs present in our assembly, between 220,164 (72 h, rep4) and 292,399 (24 h, rep 2) primary contigs (components) were represented among the 15 libraries (Additional file 1: Table S1). Among all primary contigs, 388,193 (representing 98,254 genes) were present in at least one sample. However, 164,810 contigs (representing 18,414 genes) were expressed at levels greater than 10 transcripts per million (TPM, see Additional file 2: RNA-seq master file). From these contigs, 7855 genes had differential transcript abundance (posterior probability of being differentially expressed (PPDE) ≥ 0.95) based on the EBseq program (see Methods section) of the four bud sampling time points (0, 6, 24, and 72 h). Principal component analysis of these 7855 genes revealed similarities and differences between the physiological states (Fig. 2). The first dimension of the analysis, the X-component, explained 68 % of the variance and clearly distinguished 24 h growth-induced buds from other time points (0 h, 6 h, and 72 h). The Y-component explained 17 % of the variance, indicating that the physiological state of the 72 h buds was similar to both 0 h and 6 h buds, whereas 0 h and 6 h buds were not as similar to each other as to 72 h buds (higher Y variance). Nevertheless, principal component analysis clearly separated these 4 groups of buds, indicating divergent physiological states among them.

**Fig. 3:**
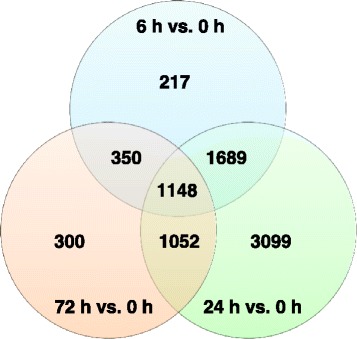
Venn diagram showing the distribution of differentially-expressed genes that are unique or common among three comparisons: 6 h vs. 0 h, 24 h vs. 0 h, and 72 h vs. 0 h

Using paradormant (0 h) buds as a baseline, statistical analyses were performed to compare buds from various growth-induced time points; i.e., 6 h vs. 0 h, 24 h vs. 0 h, and 72 h vs. 0 h. Analysis indicated 3404, 6988, and 2850 genes had differential transcript abundance for the 6 h vs. 0 h, 24 h vs. 0 h, and 72 h vs. 0 h comparisons, respectively. The distribution of genes associated with transcripts that are unique and common among three comparisons is shown in the Venn diagram (Fig. 3 and Additional file 2: RNA-seq master file – Pattern key). The results indicate that 217 out of 3404 genes with differential transcript abundance were unique for 6 h vs. 0 h, 3099 out of 6988 were unique for 24 h vs. 0 h, and 300 out of 2850 were unique for 72 h vs. 0 h. The 3099 unique gene set for 24 h vs. 0 h supports the results obtained in principal component analysis (Fig. 2) that the physiological states of 24 h growth-induced buds were most dissimilar among 4 time points. There were 1689 genes common in transcript abundance between 6 h vs. 0 h and 24 h vs. 0 h, 1052 common between 24 h vs. 0 h and 72 h vs. 0 h, 350 common between 72 h vs. 0 h and 6 h vs. 0 h, and 1148 common among the three comparisons.

### RT-qPCR

**Fig. 4:**
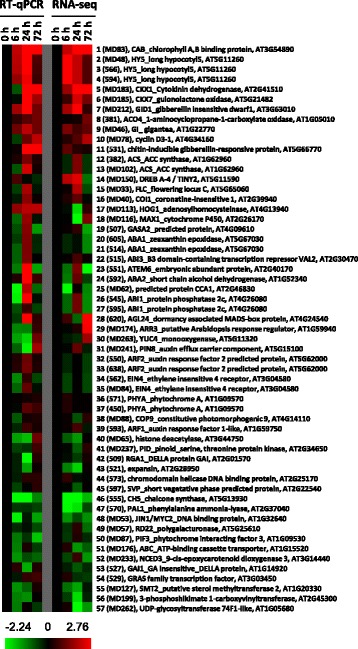
Heat map diagram showing changes in gene expression obtained by RT-qPCR vs. RNA-seq analysis. Each column represents a treatment starting from paradormant control buds (0 h) to buds at 6, 24, and 72 h post-shoot removal. Fold difference in transcript abundance is designated as log2. Red color indicates up-regulated genes and green color indicates down-regulated genes as compared to control, which was set to zero (black). The primer pair number for RT-qPCR is shown within the parentheses

RT-qPCR was used to validate the transcriptomics data obtained from RNA-seq. Fifty seven genes involved in growth, hormone, light, and temperature response/regulation (Fig. 4 and Additional file 3: Table S2) were examined. The results demonstrate that transcript abundance generated by RT-qPCR and RNA-seq was very similar (Fig. 4, see also Additional file 3: Table S2 for numeral values). Overall, correlation analysis between RNA-seq and RT-qPCR expression analyses for this set of selected genes indicated that the 6 h, 24 h and 72 h time points had a correlation coefficient of 0.78, 0.61, and 0.80 respectively. In addition, the expression intensity appears similar between these two systems. For example, 6 h, 24 h, and 72 h after paradormancy release, the increased folds (based on log2) in transcript abundance of a putative leafy spurge *CHLOROPHYLL A/B-BINDING PROTEIN* (*CAB*) were 0.91, 2.29, and 2.63 for RT-qPCR and 0.93, 1.63, 2.17 for RNA-seq (Fig. 4, #1), respectively. The increased abundance of *CAB* transcript was among the fastest responses observed and reflected the bud’s prompt photosynthetic response to perceiving a growth-inducing signal. Similar observation also applies to decreased folds in transcript abundance for a putative leafy spurge *CHALCONE SYNTHASE* (*CHS*), which were −2.24, −1.78, and −0.64 for RT-qPCR and −1.79, −2.22, and −0.87 for RNA-seq (Fig. 4, #46), respectively.

The differential abundance of other transcripts correlated well with the physiological status for crown buds after paradormancy release. The abundance of a putative *ELONGATED HYPOCOTYL 5* (*HY5*) transcript increased between 6 and 24 h after paradormancy release (Fig. 4, #2, #3, & #4). In *Arabidopsis*, HY5 is a bZIP transcription factor required for photomorphogenesis and is regulated by crosstalk between GA and the CONSTITUTIVE PHOTOMORPHOGENESIS1 ubiquitin pathway [39]. The transcript profile of *HY5* was very similar to that of *CAB* mentioned above. These results suggest that signaling mechanisms involved in paradormancy release may also play a role in activation of photosynthetic machinery. In accordance with this observation, the abundance of transcript with similarity to a GA receptor, *GIBBERELLIN INSENSITIVE DWARF1* (*GID1*), increased 6 h after paradormancy release, and reached peak levels at 24 h time point (Fig. 4, #7). The abundance of a transcript with similarity to *GA INSENSITIVE1* (*GAI1*) (a negative regulator of the GA signaling pathway) decreased 24 h after paradormancy release (Fig. 4, #53). In addition, abundance of transcripts with similarity to cell division related genes, *CYTOKININ OXIDASE 1* (*CKX1*), *CKX7*, and *CYCLIN D3-1* (*CYCD3-1*), increased 24 through 72 h after paradormancy release (Fig. 4, #5, #6, & #10); in contrast, a transcript similar to an ABA biosynthetic gene, *9-CIS-EPOXYCAROTENOID DIOXYGENASE 3* (*NCED 3*), decreased 6 h and 72 h after paradormancy release (Fig. 4, #52). These data indicate that distinct cellular responses occurred during the transition from paradormancy to shoot-induced growth.

### Differential abundance of hormone-related transcripts is overrepresented

**Fig. 5:**
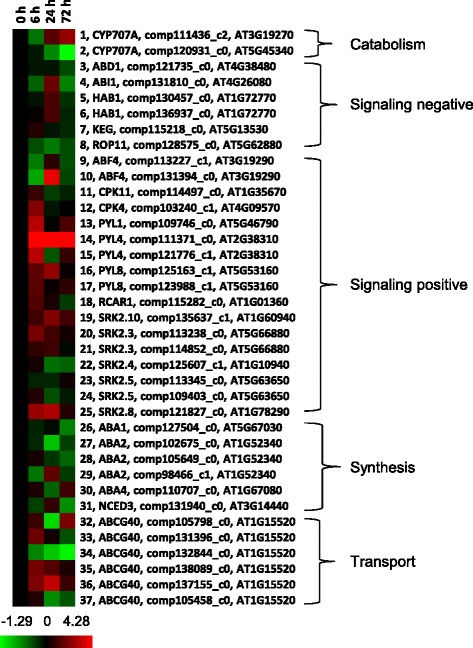
Profile of ABA-related transcripts obtained from crown buds of leafy spurge between 0 and 72 h post-shoot removal. Fold difference in transcript abundance is designated as log2, which is the average of 3 or 4 biological replicates. Red color indicates up-regulated genes and green color indicates down-regulated genes as compared to 0 h control, which was set to zero (black)

Since phytohormones play critical roles in the regulation of bud growth and development, the abundance of hormone-related transcripts were determined using the RNA-seq data. There were 373 transcripts annotated as hormone-related genes (genes with known roles in synthesis, catabolism, transport, or direct positive or negative signaling roles). Of these, 185 had differential transcript abundance and were significantly over-represented (*p* = 0.001) (Table 1 and Additional file 4: Table S3). Transcripts associated with ABA were most over-represented with a hypergeometric *p*-value of 0.009 (Table 1). Of the 6 transcripts associated with negative ABA signaling (Fig. 5, #3 to #8), three had peak abundance at 24 h and 5 had minimum abundance at 72 h after paradormancy release. Also, a majority of the 17 transcripts associated with positive ABA signaling (Fig. 5, #9 to #25) had the greatest abundance at 6 h after paradormancy release, although no obvious pattern was observed for the timing of minimum abundance. This observation indicated a shift in ABA levels and/or signals during these three time points. Among the 6 putative ABA synthesis-encoding genes (Fig. 5, #26 to #31), most had decreased transcript abundance at 6–72 h compared to paradormant buds, whereas 4 of the 6 putative ABA transport-encoding transcripts (Fig. 5, #32 to #37) had maximum abundance at the 6 to 24 h.

**Table 1:** Hypergeometric distribution of over-represented hormone-related genes

Hormone related genes	Total population size	Significant population size	*p* value
Total number	373	185	0.001
ABA	66	37	0.009
Auxin	97	50	0.017
BR	38	19	0.085
Cytokinin	44	20	0.112
Ethylene	22	12	0.090
GA	28	12	0.151
JA	55	25	0.099
SA	23	10	0.166
total	18,415	7855	1

**Fig. 6:**
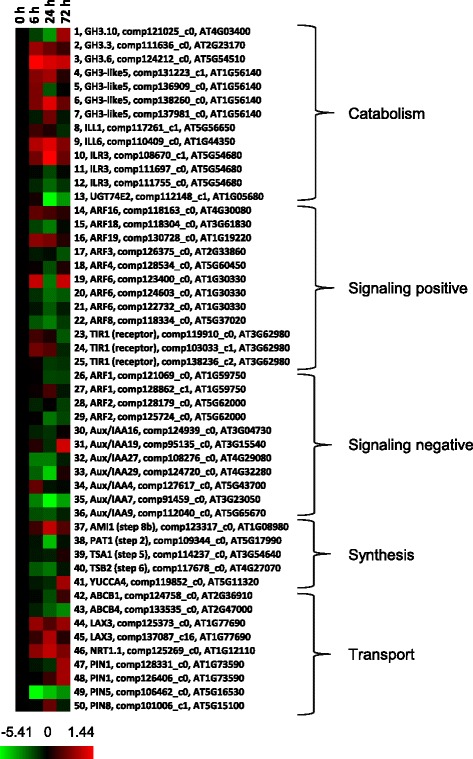
Profile of auxin-related transcripts obtained from crown buds of leafy spurge between 0 and 72 h post-shoot removal. Fold difference in transcript abundance is designated as log2, which is the average of 3 or 4 biological replicates. Red color indicates up-regulated genes and green color indicates down-regulated genes as compared to 0 h control, which was set to zero (black)

Auxin was the second most over-represented with a hypergeometric *p*-value of 0.017 (Table 1). Among 13 transcripts associated with auxin catabolic process (Fig. 6, #1 to #13), most had low abundance in paradormant UABs (0 h time point) compared to other time points, and 4 of the 5 transcripts associated with auxin synthetic process (Fig. 6, #37 to #41) were less abundant at the 24 h time point compared to paradormant UABs. Although no strong patterns were observed for transcripts associated with positive regulation of auxin signaling, all three transcripts with similarity to auxin receptor-encoding genes (TIR1s; Fig. 6, #23 to #25) had increased abundance at the 6 h time point after paradormancy release. Of the 11 transcripts with similarity to negative regulators (Fig. 6, #26 to #36), 9 had their lowest abundance at 24 h after paradormancy release. No obvious patterns of abundance were noted for the transcripts with putative similarity to transporters.

**Fig. 7:**
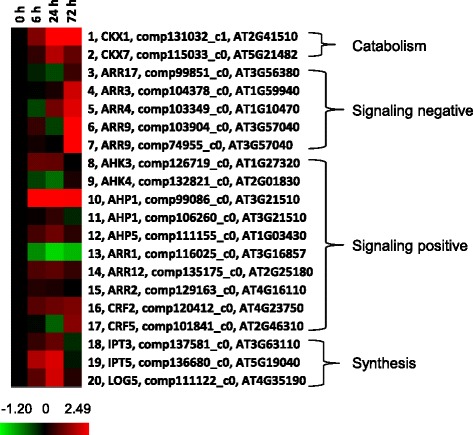
Profile of cytokinin-related transcripts obtained from crown buds of leafy spurge between 0 and 72 h post-shoot removal. Fold difference in transcript abundance is designated as log2, which is the average of 3 or 4 biological replicates. Red color indicates up-regulated genes and green color indicates down-regulated genes as compared to 0 h control, which was set to zero (black)

Transcripts associated with cytokinin levels/signaling were not significantly over-represented with a hypergeometric *p*-value of 0.112 (Table 1). However, it should be noted that transcripts associated with cytokinin catabolic processes (*CKX1 & 7*; Fig. 7, #1 and #2) and synthesis (*IPT3 & 5* and *LOG5*; Fig. 7, #18 to #20) had increased abundance after paradormancy release. The differences between them were that transcripts associated with cytokinin synthesis were less abundant at 72 h whereas transcripts associated with cytokinin catabolic processes stayed abundant. It is known that cytokinin induces multiple *CKX*s in *Arabidopsis* [40]. The concurrent increased abundance of transcripts associated with both cytokinin catabolic and synthetic processes may imply that both are needed to maintain an optimal cytokinin concentration.

Transcripts associated with GA biosynthesis/signaling processes were not over-represented (Table 1). However, it should be noted that transcripts with similarity to GA receptors (*GID1A* and *GID1B*) had peak abundance at 24 h after paradormancy release (Additional file 4: Table S3; hormone GA, #5 to #7). Among the JA-associated transcripts that also missed the 0.05 over-representation cutoff for significance (Table 1), 10 of the 14 transcripts associated with JA synthesis were highly abundant at 0 or 6 h time point and their abundance gradually decreased thereafter (Additional file 4: Table S3; hormone JA, #12 to #25).

### Gene set- and sub-network- enrichment analysis

We performed GSEA using the RNA-seq data to identify metabolic processes in crown buds during the transition from paradormancy to shoot-induced growth based on AraCyc pathways (see Methods section). GSEA determined over-represented sets of transcripts with increased or decreased abundance for comparisons 6 h vs. 0 h, 24 h vs. 0 h, and 72 h vs. 0 h. The GSEA results are summarized in Table 2 and the subsequent sections. Up and down regulated gene lists (indicated by arrows) are growth-induced (6 h, 24 h, and 72 h) compared with 0 h time point. Pathway descriptions, genes, and additional data for each comparison are available in Additional file 5: Table S4. Most pathways were among either up- or down-regulated gene lists; still, some pathways were over-represented among both up- and down-regulated gene lists. SNEA identified expression targets and small molecules as central hubs for over-represented transcripts of a given dataset. Table 3 shows expression targets and small molecules identified as central hubs for comparisons 6 h vs. 0 h, 24 h vs. 0 h, and 72 h vs. 0 h (also see Additional file 6: Table S5).

**Table 2:** AraCyc pathways that are over-represented for comparisons 6 h vs. 0 h, 24 h vs. 0 h, and 72 h vs. 0 h based on Gene Set Enrichment Analysis

AraCyc pathways	6 h vs. 0 h		24 h vs. 0 h		72 h vs. 0 h	
13-LOX and 13-HPL pathway	↑					↑
2,4,6 trinitrotoluene degradation					↑	
Abscisic acid glucose ester biosynthesis					↑	↓
Ajugose biosynthesis (galactinol-dependent)					↑	↓
Ajugose biosynthesis II (galactinol-independent)					↑	↓
Beta-alanine biosynthesis I	↑					
Brassionosteriod biosynthesis II	↑					
Calvin cycle			↑	↓		
Cellulose biosynthesis				↓		
Chlorophyll a biosynthesis II				↓		
Chlorophyllide a biosynthesis					↑	
choline biosynthesis II				↓		
Chloline biosynthesis III				↓		
Chorismate biosynthesis				↓		
Coumarin biosynthesis (via 2-coumarate)	↑		↑		↑	
Cuticular wax biosynthesis		↓				↓
Cutin biosynthesis	↑				↑	
Cyanate degradation	↑					
Cysteine biosynthesis					↑	
Cytokinins 7-N-glucoside biosynthesis					↑	↓
Cytokinins 9-N-glucoside biosynthesis					↑	↓
Cytokinins-O-glucoside biosynthesis					↑	↓
Ethylene biosynthesis from methionine				↓		↓
Fatty acid biosynthesis-initial steps		↓				
Flavonoid biosynthesis		↓		↓		↓
Flavonol biosynthesis				↓		
Galactose degradation I		↓				
Galactose degradation II (III)	↑					
Galactosylcyclitol biosynthesis					↑	↓
Gluconeogenesis	↑		↑	↓	↑	
Glucosinolate biosynthesis from dihomomethionine		↓				
Glucosinolate biosynthesis from hexahomome thionine		↓				
Glucosinolate biosynthesis from pentahomomethionine		↓				
Glucosinolate biosynthesis from phenylalanine	↑	↓				
Glucosinolate biosynthesis from tetrahomomethionine		↓				
Glucosinolate biosynthesis from trihomomethionine		↓				
Glucosinolate biosynthesis from tryptophan	↑	↓				
Glycolipid desaturation		↓				
Glycolysis I (plant cytosol)				↓		
Glycolysis II (plant plastids)				↓		
Homogalacturonan biosynthesis				↓		
Homogalacturonan degradation	↑			↓	↑	
Hydroxyjasmonate sulfate biosynthesis		↓				
IAA biosynthesis I			↑			
IAA biosynthesis II						↓
IAA biosynthesis VII		↓				
IAA degradation IV					↑	↓
Jasmonic acid biosynthesis	↑			↓		↓
Kaempferol glucoside biosynthesis (Arabidopsis)					↑	↓
Leucine degradation			↑			
Leucodelphin biosynthesis	↑		↑	↓		
Methionine biosynthesis				↓		
Methionine salvage pathway				↓		
Methylindole-3-acetate interconversion		↓		↓		↓
Methylquercetin biosynthesis				↓		
Monolignol glucosides biosynthesis					↑	↓
Oxidative ethanol degradation I			↑			
Pelargonidin conjugates biosynthesis					↑	↓
Phenylalanine degradation III			↑			
Phenylpropanoid biosynthesis	↑		↑			
Phosphatidylcholine biosynthesis IV				↓		
Phospholipases				↓		↓
Phospholipid desaturation		↓				
Photorespiration	↑		↑			
Photosynthesis	↑		↑	↓	↑	
Photosynthesis, light reaction	↑		↑		↑	
Plastoquinone-9 biosynthesis				↓		
Pyridine nucleotide cycling (plants)			↑			
Quercetin glucoside biosynthesis					↑	↓
Quercentinsulphates biosynthesis				↓		
Rubisco shunt				↓		
Salicylic acid biosynthesis	↑	↓				
SAM cycle				↓		
S-methylmethionine cycle				↓		
Sphingolipid biosynthesis (plants)						↓
Starch biosynthesis		↓		↓		↓
Starch degradation		↓				↓
Suberin biosynthesis		↓				↓
Sucrose degradation to ethanol and lactate (anaerobic)			↑	↓	↑	
Superpathway of acetyl-CoA biosynthesis				↓		
Superpathway of anthocyanin biosynthesis (from cyanidin and cyanidin3-O-glucoside)		↓				
Superpathway of choline biosynthesis				↓		↓
Superpathway of cytosolic glycolysis (plants),pyruvate dehydrogenase and TCA cycle			↑	↓		
Superpathway of fatty acid biosynthesis		↓				
Superpathway of lysine, threonine, and methionine biosynthesis				↓		
Superpathway of phenylalanine and tyrosine biosynthesis		↓		↓		
Superpathway of phenylalanine, tyrosine and tryptophan biosynthesis				↓		
Superpathway of phosphatidylcholine biosynthesis				↓		
Superpathway of plastoquinone biosynthesis				↓		
Superpathway of starch degradation to pyruvate				↓		
Superpathway of sucrose and starch metabolism I (non-photosynthetic tissue)	↑	↓		↓		↓
Superpathway of sucrose and starch metabolism II (photosynthetic tissue)		↓				
Superpathway of sucrose degradation to pyruvate				↓	↑	
Trehalose biosynthesis		↓		↓	↑	
Triacylglycerol biosynthesis		↓				
Triacylglycerol degradation	↑				↑	↓
Ubiquinone-9 bipsynthesis (eukaryotic)				↓		
UDP-D-xylose biosynthesis				↓		
UDP-sugars interconversion				↓		
Very long chain fatty acid biosynthesis		↓				
Vitamin E biosynthesis				↓		
Xylan biosynthesis					↑	
Zeaxanthin biosynthesis		↓				

Up and down arrows indicate the direction of regulation in the former part of the comparison (i.e., 6 h vs. 0 h: up means up in 6 h). Genes and additional data within each pathway for each comparison are available in Additional file 5: Table S4

**Table 3:** Expression targets and small molecules identified as central hubs for comparisons 6 h vs. 0 h, 24 h vs. 0 h, and 72 h vs. 0 h based on sub-network enrichment analyses

	Expression targets_up	Expression targets_down	Small molecules_up	Small molecules_down
6 h vs. 0 h	EIN4, EIN2, XRN4, EIN3, MYC2, COI1, CCA1, COP1	HSF, PAP1, ABI3, photoreceptor	salicylate, carbohydrates, JA, cytokinin, diuron, phytohormone, Na+, H2O, Grelutin	NO, MeJA, Cu2+, Cd2+, brassinosteroids, chitosan
24 h vs. 0 h	STM	E2F, E2F3, PAP1, basic-helix-loop- helix protein	JA, sulfur, N- Benzyladenine, H2SO4, Grelutin, L-glutamine, gibberellin	Mitomycin, carbohydrates, EGTA, anthocyanins, hydroxyurea, MeJA, Paclobutrazol
72 h vs. 0 h	EIN3, DNA- directed RNA polymerase	EIN3, ZTL, ABI1, RGA1	cytokinin, lincomycin, CO2, salicylate, NO, Geldanamycin, tunicamycin, D- glucose	ethylene, Ca2+, H2O, NaCl, NADP+, NO, D- mannitol

Genes and additional data for each comparison are available in Additional file 6: Table S5

*6 h* vs. *0 h:* Forty five AraCyc pathways were over-represented 6 h after paradormancy release (Table 2). Among them, 16 pathways were up-regulated, 25 were down-regulated, and 4 were associated with both up- and down-regulated genes. Most of the up-regulated pathways were biosysynthetic pathways, and were involved in JA (13-LOX and 13-HPL pathway), beta-alanine, BR, coumarin, cutin, glucose (gluconeogenesis), leucodelphinidin, and phenylpropanoid biosynthesis. The rest of the up-regulated pathways included photorespiration, photosynthesis, and some degradation pathways such as cyanate, galactose (galactose degradation II, III), homogalacturonan, and triacylglycerol degradation pathways. These up-regulated pathways likely imply that buds detect sudden physiological changes in response to shoot removal and prepare for growth by synthesizing new hormones and cell wall materials.

Similar to up-regulated pathways, most of the down-regulated pathways were biosynthesis pathways, and they were involved in cuticular wax, fatty acid (also include very long chain fatty acid), flavonoid, glucosinolate (total 5 groups), hydroxyjasmonate sulfate, IAA, starch, suberin, anthocyanin, phenylalanine, tyrosine, trehalose, triacylglycerol, and zeaxanthin biosynthesis. The rest of the down-regulated pathways were involved in galactose and starch degradation, glycolipid desaturation, methyl indole-3-acetate interconversion, phospholipid desaturation, and sucrose and starch metabolism II (photosynthetic tissue). The 4 pathways associated with up- and down-regulated genes included glucosinolate biosynthesis from phenylalanine, glucosinolate biosynthesis from tryptophan, salicylic acid (SA) biosynthesis, and superpathway of sucrose and starch metabolism. Many over-represented pathways at this time point (6 h) are involved in defense responses, and may have been altered due to the wounding caused by excision of the aerial shoot.

SNEA of up-regulated genes 6 h after dormancy release (Table 3) identified *ETHYLENE INSENSITIVE4* (*EIN4*), *EIN2*, *EXORIBONUCLEASE4* (*XRN4*), *EIN3*, *MYC2*, *CORONATINE*-*INSENSITIVE 1* (*COI1*), *CIRCADIAN CLOCK ASSOCIATED 1* (*CCA1*), and *CONSTITUTIVE PHOTOMORPHOGENESIS 1* (*COP1*) as central hubs for expression targets. A notable feature with these hubs is that they have been reported to play key roles in wounding responses [41, 42] and photomorphogenesis [43] in other organisms. In addition, salicylate, JA, and cytokinin were the major hubs for small molecules as judged by their number of neighbors (Table 3). Small molecules provide information about the physiological and molecular state of buds and often bind to specific receptors to initiate signaling cascades.

SNEA of down-regulated genes 6 h after dormancy release (Table 3) identified *HEAT SHOCK FACTOR* (*HSF*), *PRODUCTION OF ANTHOCYANIN PIGMENT1* (*PAP1*), *ABSCISIC ACID INSENSITIVE3* (*ABI3*), and photoreceptors as central hubs for expression targets, and the major hubs for small molecules were MeJA and NO. PAP1, also called MYB75, is a regulator of the anthocyanin branch of the phenylpropanoid pathway and secondary cell wall formation in *Arabidopsis* [44].

*24 h* vs. *0 h:* Fifty five AraCyc pathways were over-represented 24 h after paradormancy release (Table 2). Among them, 9 pathways were up-regulated, 40 were down-regulated pathways, and 6 were associated with both up- and down-regulated genes. Up-regulated pathways include coumarin, IAA, and phenylpropanoid biosynthesis; leucine, oxidative ethanol, and phenylalanine degradation; photorespiration; photosynthesis; and pyridine nucleotide cycling (plants). A notable feature among up-regulated pathways is that IAA biosynthesis pathway (IAA biosynthesis I) was up-regulated at this time point.

Among 40 down-regulated pathways, most of which were involved in biosynthesis, and these were cellulose, chlorophyll a, choline, chorismate, ethylene, flavonoid, flavonol, homogalacturonan, JA, methionine, methylquercetin, phosphatidylcholine, plastoquinone(−9), quercetinsulphates, starch, acetyl-CoA, choline, lysine, threonine, phenylalanine, tyrosine, tryptophan, phosphatidylcholine, trehalose, ubiquinone-9, UDP-D-xylose, and vitamin E biosynthesis. The rest of down-regulated pathways included homogalacturonan degradation, starch degradation to pyruvate, sucrose degradation to pyruvate, glycolysis I and II, methionine salvage, methyl indole-3-acetate interconversion, phospholipases, rubisco shunt, SAM cycle, S-methylmethionine cycle, sucrose and starch metabolism, and UDP-sugars interconversion. The large numbers of down-regulated pathways relative to up-regulated pathways is notable. Pathways associated with up- and down-regulated included Calvin cycle, gluconeogenesis, leucodelphinidin biosynthesis, photosynthesis, sucrose degradation to ethanol and lactate, and superpathway of cytosolic glycolysis, pyruvate dehydrogenase and TCA cycle. Most of these pathways are related to carbon and energy use.

SNEA of up-regulated genes 24 h after paradormancy release (Table 3) identified only one central hub, *SHOOT MERISTEMLESS* (*STM*), for expression targets, which may be associated with cell proliferation. The major hubs for small molecules were JA and GA. SNEA of down-regulated genes 24 h after dormancy release (Table 3) identified *E2F*, *E2F3*, *PAP1*, and basic-helix-loop-helix (bHLH) protein. The major hubs for small molecules were MeJA, carbohydrates, and anthocyanins.

*72 h* vs. *0 h:* Forty AraCyc pathways were over-represented 72 h after paradormancy release (Table 2). Among them, 13 pathways were up-regulated, 14 were down-regulated pathways, and 13 were up- and down-regulated. Most of the up-regulated pathways were biosysynthetic pathways, and were involved in chlorophyllide a, coumarin, cutin, cysteine, glucose, trehalose, and xylan biosynthesis. The rest of the up-regulated pathways were photosynthesis and several degradation pathways such as 2,4,6-trinitrotoluene, homogalacturonan, and sucrose degradation. The notable feature among up-regulated pathways is that they were involved in growth and development. Most of the down-regulated pathways were also involved in biosynthesis, and they were JA (13-LOX and 13-HPL pathway), cuticular wax, ethylene, flavonoid, IAA, sphingolipid, starch, suberin, and choline biosynthesis. The rest of the down-regulated pathways were methyl indole-3-acetate interconversion, phospholipases, starch degradation, and sucrose and starch metabolism. The notable feature of these pathways is that many hormone biosynthetic pathways were down-regulated. Up- and down-regulated pathways included many biosynthesis pathways such as ABA glucose ester, ajugose (galactinol-dependent and galactinol-independent), cytokinins 7-N-glucoside, cytokinins 9-N-glucoside, cytokinins-O-glucoside, galactosylcyclitol, kaempferol glucoside, monolignol glucosides, pelargonidin conjugates, and quercetin glucoside biosynthesis pathways, and two degradation pathways that were involved in IAA and triacylglycerol degradation. Overall, these pathways reflected that activities for various phytohormones were altered at this time point.

SNEA of up-regulated genes 72 h after dormancy release (Table 3) identified only *EIN3* and DNA-directed RNA polymerase central hubs for expression targets. The major hubs for small molecules were salicylate, cytokinin, D-glucose (Table 3). SNEA of down-regulated genes at this time point identified *EIN3*, *ZEITLUPE* (*ZTL*), *ABI1*, and *RGA1* (Table 3). *EIN3* was also identified as a central hub of transcripts with increased abundance (see above). The major hubs for small molecules were ethylene, NaCl, and Ca^2+^. Overall, SNEA suggests that hormone and light signaling were altered when buds initiated growth.

### Phytohormone levels after paradormancy release

**Fig. 8:**
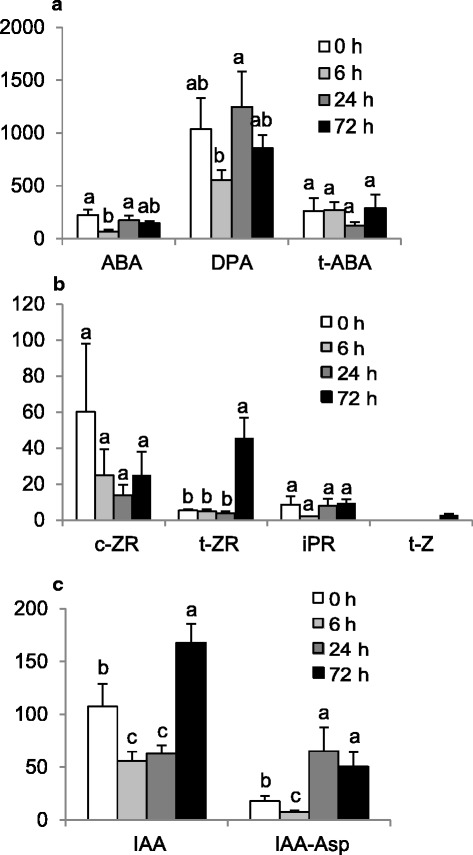
Profiles of ABA (**a**), cytokinin (**b**), and IAA (**c**) levels measured from crown buds of leafy spurge between 0 and 72 h post-shoot removal. These profiles represent the average of four biological replicates ± SE. Means labeled by the same letter are not significantly different (*P* < 0.1)

ABA, cytokinins, auxins, and GA levels were measured in paradormant crown buds before and after paradormancy release (Fig. 8). Among 4 time points, ABA levels were greatest in paradormant buds (0 h); ABA content of these buds was 221 ng g^−1^ DW (dry weight). Six h after paradormancy release, ABA content dropped to 65 ng g^−1^ DW and then increased to 174 ng g^−1^ DW between 6 and 24 h, after which ABA content diminished again towards 72 h. ABA metabolite dihydrophaseic acid (DPA) contents were relatively high in bud samples but had similar trends in concentration as that of ABA; the greatest DPA level (1245 ng g^−1^ DW) was observed at 24 h, and the least (552 ng g^−1^ DW) was at 6 h. Trans-ABA (*t*-ABA) levels, a product of isomerization of natural ABA under UV light, did not show significant changes after paradormancy release (Fig. 8a).

The levels of most biologically active free base cytokinins were too low to allow reliable measurement; only *trans*-zeatin (*t*-Z) was detected definitively 72 h after paradormancy release, and it was present in a small amount (3 ng g^−1^ DW). However, the levels of their biosynthetic precursors, *cis*-zeatin riboside (*c*-ZR) and *trans*-zeatin riboside (*t*-ZR), were relatively high in crown bud samples. While the levels of *c*-ZR were not significantly different due to high variations in the biological replicates of paradormant buds, *t*-ZR levels increased dramatically between 0 h to 72 h at 5 and 46 ng g^−1^ DW, respectively. Another biosynthetic precursor, isopentenyladenine riboside (iPR), did not show significant changes after paradormancy release (Fig. 8b).

Auxins are mainly represented by biologically active indole-3-acetic acid IAA and its conjugates with aspartic acid N-(indole-3-yl-acetyl)-aspartic acid (IAA-Asp) [21]. Our results indicated that IAA levels were relatively high (107 ng g^−1^ DW) in paradormant buds and declined almost 2-fold 6 h after shoot removal (56 ng g^−1^ DW). It appeared that buds synthesized IAA after the transition from dormancy to growth since IAA levels increased dramatically from 24 to 72 h at 63 and 168 ng g^−1^ DW, respectively. IAA-Asp levels also declined from 0 to 6 h at 18 and 7 ng g^−1^ DW, respectively. IAA-Asp had a constant increase towards 24 h (65 ng g^−1^ DW) and 72 h (50 ng g^−1^ DW), at levels significantly higher than that of 0 h time point (Fig. 8c).

We also attempted to measure GA levels, and traces of GA3, GA19, and GA24 were detected at later time points but could not be reliably quantified (data not shown). Nevertheless, our results suggest that GA levels were generally low but were increasing as UABs transitioned from paradormancy to shoot-induced growth.

## Discussion

This study compared phytohormone abundance and the transcriptomes of paradormant UABs vs. shoot-induced growth at 6, 24, and 72 h after paradormancy release based on hormone profiling and RNA-seq analyses. The assembled transcriptome was annotated against the nonredundant and TAIR *Arabidopsis* database. The expression data (in transcripts per million) were further subjected to principal component analysis and gene set- and sub-network- enrichment analysis. The results showed that differential abundance of transcripts associated with hormone signaling, the high number of over-represented ontologies associated with specific phytohormones or hormone processes, and the concurrent changes in phytohormone levels are all well correlated. Combined, these observations suggest that signals induced by the loss of the aerial shoots altered phytohormone abundance/perception and led to transcriptome changes, which facilitated cellular changes requisite for paradormancy release and differentiation to shoot growth.

### ABA, IAA, and flavonoids appear to maintain paradormancy in UABs

Hormones, particularly auxin and ABA, have long been associated with regulating bud outgrowth following loss of growing shoot apices [12] and contributing to paradormancy maintenance in vegetative buds [27, 45]. Thus, it was not surprising to find that these hormones were implicated with maintenance of paradormancy in this study and support the validity of our transcriptome results. Relatively high levels of ABA were found in paradormant buds (0 h, before removal of aerial shoots) (Fig. 8a). The decrease in ABA levels after paradormancy release seems consistent with the abundance of ABA-related transcripts. For example, a transcript associated with ABA biosynthesis, *NCED3* (Fig. 4, #52 and Fig. 5, #31), was less abundant 6 h after paradormancy release (Fig. 5). NCED is involved in catalyzing the rate-limiting step in ABA biosynthesis [46]. Similar results were also obtained for other transcripts involved in ABA biosynthesis such as *ABA DEFICIENT1*, *2*, & *4* (*ABA1*, *2*, & *4*; Fig. 5, #26 to #30). Although ABA biosynthesis appeared to be decreased at the 6 h time point, as indicated by the significant reduction of ABA and DPA (Fig. 8a), abundance of transcripts involved in ABA catabolism (cytochrome P450 *CYP707A1* and *CYP707A4*) were less abundant 6 h after paradormancy release (Fig. 5, #1 and #2). In *Arabidopsis*, *CYP707A* encodes ABA 8′-hydroxylases, which catalyze the hydroxylation of ABA at the C-8′ to form unstable 8′-hydroxy ABA molecules [47].

Abscisic Acid Responsive Elements (ABREs) are the major *cis*-regulatory element for ABA-responsive gene expression, and ABRE-binding factors (ABFs) are transcription factors that regulate ABRE-dependent gene expression. In *Arabidopsis*, *ABF4* can be induced by dehydration, high salinity and ABA treatment in vegetative tissues [48]. Two putative leafy spurge *ABF4* transcripts were less abundant in crown buds of leafy spurge at the 6 h time point (Fig. 5, #9 and #10). Because ABA is known to inhibit bud growth [9] and some putative *ABF4* transcripts were less abundant during paradormancy release, it appears that ABA and ABA-related signaling may play an important role in maintenance of paradormancy in UABs of leafy spurge.

Relatively high levels of IAA were also observed in paradormant buds (Fig. 8c). Higher auxin levels in paradormant buds are consistent with the conventional view that IAA is the key factor for paradormancy maintenance [49–51]. Still, this observation is somewhat surprising given that auxin is generally produced more in growing shoot tips rather than in dormant buds [52]; in addition, the current dogma indicates that once buds are released from paradormancy, bud outgrowth should be accompanied by increased auxin production and export [23, 24]. High auxin levels prior to paradormancy release might result from low auxin export because putative auxin transporters *LAX3* (Fig. 6, #44 and #45), *NRT1.1* (#46) and *PIN1* (#47 and #48) all had low baseline transcript abundance at 0 h compared to later time points. Similarly, of the 9 transcripts with differential abundance for transporters (Fig. 6. #42 to #50), only *ABCB4* (#43) and *PIN5* (#49) had a significant decrease at 6–72 h compared to 0 h.

*GH3* genes are auxin-inducible and encode enzymes that catalyze IAA conjugates (inactive forms) to control the intracellular IAA level through a homeostatic feedback regulatory loop [21]. Abundance of putative transcripts to *GH3* (Fig. 6, #1 to #7) were, in general, greater 6 h after paradormancy release with the exception of #1 and #7. Thus, reduced auxin levels following paradormancy release might be due to a feedback regulation in auxin production. Alternatively, the chosen time points were too early to detect an expected increase in auxin production or that basal auxin production is sufficient to maintain the required export needed to ensure bud outgrowth.

Perhaps a more intriguing observation is the rapid decrease in abundance of transcripts involved in the flavonoid biosynthesis pathway after paradormancy release (Table 2). A transcript (*CHS*) involved in flavonoid biosynthesis had relatively high baseline abundance in paradormant buds (0 h) compared to the other three time points (Fig. 4, #46). Because flavonoids are known to inhibit auxin transport [53], these results could suggest that increased levels of cellular flavonoids act to inhibit bud growth by impinging on auxin transport. This hypothesis is consistent with aforementioned findings that transcripts for putative auxin transporters were less abundant in paradormant buds. These results are also consistent with previous studies that implicated a similar response during paradormancy release [38].

### Paradormancy release caused rapid alteration of phytohormone profiles

Within 6 h after paradormancy release, a sharp drop in ABA and IAA levels was observed (Fig. 8a and c). This sudden physiological change did not appear to be a stress response, since stress generally stimulates ABA biosynthesis [10]. At the molecular level, cellular responses were also consistent with decreased ABA levels or signaling; for example, the down-regulation of the zeaxanthin biosynthesis pathway (Table 2). A transcript with similarity to an *Arabidopsis* ABA biosynthetic gene (*ABA1*) also decreased its abundance (Fig. 4, #20 & #21). *ABA1* encodes zeaxanthin epoxidase, which plays a role in the epoxidation of zeaxanthin to antheraxanthin and all-trans-violaxanthin in the ABA biosynthetic pathway. Correlated with these results, SNEA identified putative *ABI3* as a central hub for expression targets of transcripts with decreased abundance (Table 3). It is important to note that ABI3 is a transcription factor similar to maize VP1 [54], which positively regulates ABA signaling [55].

In contrast, the 13-LOX and 13-HPL pathway and the JA biosynthesis pathway, in general, appeared to be up-regulated at the 6 h time point (Table 2). In addition, SNEA identified two JA-related central hubs, *MYC2* and *COI1*, for expression targets (Table 3) among transcripts with increased abundance. MYC2 is a versatile basic helix-loop-helix (bHLH) transcription factor that, in *Arabidopsi*s, regulates JA signaling and crosstalk with other phytohormone signaling pathways such as ABA, SA, GA, and auxin [56]. COI1 is an F-box protein and component of the SCF^COI1^ complex that targets JASMONATE-ZIM DOMAIN proteins (a negative regulator of JA signaling) for ubiquitination and proteasome degradation in other species [57], and plays an important role in control of jasmonate-regulated plant development and defense [58]. Because JA has been associated with wounding responses in plants [41], the up-regulation of these JA-related pathways and central hubs could indicate JA synthesis and/or signaling was enhanced at the 6 h time point; potentially, as part of a wounding-induced defense response.

SNEA of up-regulated genes also identified ETHYLENE INSENSITIVE2 (*EIN2*), *EIN3*, and *EIN4* as central hubs for expression targets at 6 h (Table 3). EIN4 is a membrane receptor that binds to ethylene through its N-terminal domain. EIN2, also a membrane protein, regulates the accumulation of a key transcription factor EIN3, which in turn activates many downstream inducible genes in the ethylene signaling pathway in *Arabidopsis* [59, 60]. Similar to JA, ethylene has also been associated with wounding and defense responses in plants [41]. The up-regulation of these hubs may indicate increased levels of ethylene and/or ethylene signaling at this time point, possibly due to the excision of the aerial plant tissues. However, it should be noted that these same signals could be important for subsequent downstream signaling caused by paradormancy release.

Besides the above-mentioned hormone related pathways and genes, salicylate, JA, and cytokinin were also identified as major hubs of small molecules. Moreover, SA and BR biosynthesis pathways and pathways related to cell wall development were over-represented at 6 h time point. The SA pathway is often associated with defense response in plants, and cytokinin and BR are known to regulate plant growth and development [61]. Overall, it appears that 6 h after removal of aerial shoot tissues, UABs sensed and responded to a variety of signals including wounding and defense, which induced a myriad of pathways impacting hormones such as JA, ethylene, SA, and BR; these hormones, in turn, likely acted to stimulate growth response signals.

More recently, Mason et al. showed that redistribution of sugar to the axillary buds following loss of the growing shoot apices was associated with initiation of bud outgrowth in pea [62]. In addition, Kebrom and Mullet [63] showed that leaf-derived metabolic factors such as sucrose played critical role in sorghum tiller bud outgrowth. Their results differed greatly from the findings in paradormant UABs of leafy spurge where sucrose appeared to inhibit bud growth. Chao et al. [32] demonstrated that both glucose and sucrose caused suppression of UAB growth at concentrations of 30 mM. They further determined that UABs of intact paradormant plants contained the highest level of starch (32.4 ± 0.85 mg g^−1^ fresh weight [fwt]) and sucrose (9.41 ± 0.11 mg g^−1^ fwt) compared to UABs harvested 1, 3, and 5 day after decapitation; sucrose levels were all around 5 mg g^−1^ fwt after shoot removal. In contrast, fructose levels increased dramatically during bud growth, and a 3.5 and 7.6-fold increase in fructose level was observed at day 3 and 5, respectively, after shoot removal [32]. The discrepancies in the function of sugar reported in pea and sorghum [62, 63] and this study could be due to differences in the physiology of these buds – axillary and tiller buds vs. UABs and/or due to species-specific effects. Nevertheless, previous carbohydrate measurements obtained from UABs did not include a 6 h time point, which may be needed to properly address the interplay between hormones and carbohydrates.

The down regulation of starch biosynthesis (Table 2) is consistent with findings that UABs of intact leafy spurge plants contained the highest level of starch, which decreased quickly after shoot removal [32]. Although sugar levels in the paradormant UABs of leafy spurge generally had a negative impact on bud outgrowth, differential abundance for a large number of putative sugar transporters was observed following shoot removal. Indeed, of the 24 putative sugar transporters with differential abundance, 17 had increased abundance and 10 of those had the greatest abundance 6 h after paradormancy release (Additional file 7: Table S6). These observations imply that dynamic transporter activity could occur at this time point. Thus, we hypothesized that sugar molecules generated from starch degradation and/or other processes were transported across cell membranes and quickly metabolised after shoot removal.

### Transition from paradormancy to growth at 24 h may be the result of stress responses

Both principal component analysis (Fig. 2) and Venn diagram (Fig. 3) clearly set apart 24 h growth-induced buds from other groups. These results suggest that major cellular changes occurred 24 h after removal of the aerial shoot tissues. Although not obvious from the list transcripts showing differential abundance, GSEA indicated that the IAA biosynthesis pathway (IAA biosynthesis I) was up-regulated at this time point (Table 2). However, IAA levels were still low at the 24 h time point compared to paradormant buds (63 vs. 107 ng g^−1^ DW) (Fig. 8c). Therefore, although the activity of IAA biosynthesis appeared to increase at this time point, IAA levels were not significantly increased until 72 h post paradormancy release (Fig. 8c).

The increase in IAA biosynthesis activity at 24 h may be associated with the production of reactive oxygen species (ROS, a stress response) since auxin has been regarded as an intermediate to function between stress and growth responses. It is known that mild oxidative stresses mimic auxin stimuli in somatic embryogenesis [64]. In addition, mild stress in a whole plant generates phenotypical changes similar to a 2,3,5-triiodobenzoic acid (TIBA, an inhibitor of polar auxin transport)-like disturbance of auxin distribution and enhances auxin-dependent growth cycle reactivation [65, 66]. Based on these findings, up-regulation of IAA biosynthesis pathway may indicate an enhancement of auxin-dependent growth response due to shoot removal triggered stress.

Interestingly, two transcripts similar to ABA biosynthesis genes in *Arabidopsis* (*ABA2* and *NCED3*; Fig. 5, #29 and #31) and two ABRE-binding factors (*ABF4*; Fig. 5, #9 and #10) were up-regulated only at the 24 h time point compared to the 6 and 72 h time points. Thus, this result could reflect a latent stress response in buds caused by paradormancy release, which may interact with auxin signaling networks to stimulate an auxin-dependent growth response [65, 66]. This result correlates well with the down-regulation of 40 over-represented AraCyc pathways and reflects that the stress-derived cellular responses were most evident at this time point. In relation to this observation, past experiments indicate a transient repression of growth and cell cycle genes at 24 h after excision of the aerial portion of the plant [31], which has been linked to reduction in sugar levels resulting from loss of leaf tissue and probable cross-talk between sugar and ABA signaling [32]. Our results would support these previous hypotheses.

Corroborating this notion, SNEA of transcripts with increased abundance at 24 h after dormancy release (Table 3) identified *STM* as a central hub for expression targets. *STM* encodes a class I KNOTTED-like protein that is required for shoot apical meristem (SAM) formation in *Arabidopsis* [67]. STM represses GA biosynthesis [68], and the expression of *STM* is repressed by high GA levels; in contrast, *STM* expression is induced by cytokinin, and STM promotes cytokinin biosynthesis in the SAM [69, 70]. Thus, if the products of these transcripts performed crucial functions in UABs of leafy spurge as in other plant systems, the identification of *STM* as a hub of transcripts with increase abundance at the 24 h time point indicated the initiation of cell proliferation and shoot growth. Previous studies with leafy spurge also indicated that *STM* was up-regulated 8 h following excision of the aerial portion of the plant [71]. Nevertheless, the identification of GA as a major small molecule hub among transcripts with increased abundance (Table 3) suggested that this notion may be involved in a complex signaling network at the SAM.

### Cytokinin, auxin, and GAs are required for bud growth 72 h post shoot removal

Cellular responses observed at 72 h post decapitation generally suggested that paradormant UABs had initiated the process of differentiating into shoots. Endogenous cytokinin and auxin levels increased (Fig. 8b and c), and ABA levels decreased at this time point (Fig. 8a). Several cytokinin conjugate biosynthesis pathways, cytokinins 7-N-glucoside, cytokinins 9-N-glucoside, cytokinins-O-glucoside, were up- and down-regulated (Table 2), presumably resulting in the increase of cytokinin levels (Fig. 8b). In contrast, JA-related pathways (13-LOX, 13-HPL and JA) and ethylene biosynthesis pathways were down regulated (Table 2). Both JA and ethylene are generally considered to inhibit plant growth. High levels of JA also antagonize the biosynthesis of GA in wild tobacco (*Nicotiana attenuate*) [72]. Abundance of a transcript similar to an auxin biosynthesis gene, *YUCCA flavin monooxygenase 4* (*YUCCA4*), increased at the 72 time point (Fig. 6, #41), which may indicate the involvement of auxin in the formation of vascular tissues [73]. However, the down-regulation of IAA biosynthesis II pathway was unexpected and appeared to contradict the results of increase in IAA levels (Fig. 8c). This result could be due to re-establishment of paradormancy in the more distal buds that were harvested or the possibility that the activity of IAA biosynthesis was similar or lower for 72 h buds compared to paradormant (0 h) buds. In the latter case, IAA accumulation might be a balance between biosynthesis pathway and degradation pathway, which were both up- and down-regulated at this time point (Fig. 6, #1 to #13 and #37 to #41). Alternatively, IAA biosynthesis II pathway might not be the major conduit for IAA biosynthesis in leafy spurge. Other growth related biosynthesis pathways such as chlorophyllide a, cutin, glucose, trehalose, and xylan biosynthesis pathways were all up-regulated consistent with the physiological status of these buds (Table 2).

SNEA identified *EIN3* as a central hub for expression targets of transcripts with both increased and decreased abundance (Table 3). EIN3, a transcription factor, is a positive regulator of ethylene response that regulates the expression of its downstream genes such as *ETHYLENE RESPONSE FACTOR1* [74]. Based on this observation, along with the down-regulation of ethylene biosynthesis pathway mentioned above, we postulate that ethylene biosynthesis was negatively regulated to reduce ethylene levels at this time point.

SNEA also identified *RGA1* as a hub for expression targets (Table 3) among transcripts with decreased abundance. RGA1 is a member of the DELLA regulatory family that represses the GA signaling pathway [75]. Down regulation of *RGA1* hub could suggest an increase in GA levels, which is consistent with RGA1’s negative role for GA biosynthesis. Overall, GSEA and SNEA suggested that hormone levels were altered when buds initiated growth, namely, an increase in cytokinin, auxin, and GA levels and decrease in ABA, JA, and ethylene levels.

## Conclusions

Our transcript and hormone profiling indicate that auxin, ABA, and flavonoid signaling appear to be involved in maintaining paradormancy in underground adventitious crown buds of leafy spurge, which is consistent with previous findings in underground vegetative buds of Canada thistle [45]. After paradormancy release by shoot removal, the balance of different phytohormones shifted rapidly and correlated well with differentiation of bud to shoot growth. Six h after paradormancy release, auxin, ABA, and flavonoid levels/signals were decreased, in conjunction with up-regulation of GA, cytokinin, JA, ethylene, and BR levels/signals. Assuming the transcripts identified in this study perform the same functions as they do in other plant systems, our results suggested these phytohormone signals may regulate genes affecting cell differentiation and defense responses. Twenty four h after paradormancy release, auxin and ABA levels/signals were increased, in conjunction with up-regulation of GA levels/signals. Increase in auxin and ABA levels/signals and the down-regulation of 40 over-represented AraCyc pathways may indicate that the stress-derived cellular responses were most evident at this time point, which could activate stress induced re-orientation of growth [65, 66]. Seventy two h after paradormancy release, auxin, cytokinin, and GA levels/signals were increased, whereas ABA, JA, and ethylene levels/signals were decreased. These results may suggest that UABs at this time point had recovered from stress responses and initiated normal shoot growth processes. In addition, since ABA signaling genes are negative regulators of photomorphogenesis [76], decrease in ABA level/signaling could activate rapid photomorphogenesis and in turn promote shoot growth and development.

## Methods

### Plant material

Leafy spurge UABs were prepared according to Doğramacı et al. [35, 36]. Briefly, leafy spurge plants were propagated from a uniform biotype in cone-tainers and maintained in a greenhouse [77]. This biotype (designated as ‘1984-ND-001’) was collected from a site adjacent to Hector airport, Fargo, North Dakota in 1984 [78]. Prior to the start of each experiment, plants were acclimated in a growth chamber for 1 week at 27 °C, 16:8 h light:dark photoperiod. To induce UAB growth into new shoots, all above ground shoots were excised from paradormant plants, and UABs were maintained at 27 °C, 16:8 h light:dark photoperiod in cone-tainers. Crown buds were harvested 0 h, 6 h, 24 h, and 72 h after removal of the aerial shoot tissue. Each time point had 4 replicates (reps) and each rep used about 30 plants. Hormone profiling and RT-qPCR studies included 4 reps/time point, a total of 16 samples; however, RNA-seq studies included only 3 reps for the control (0 h) and 4 reps for the remainder time points (6 h, 24 h, and 72 h), a total of 15 samples. All samples were collected around noon to avoid diurnal variation. The plants for 6 h time point were decapitated early in the day (6 AM), and UABs were collected at the same time of day as the 0, 24 and 72 h time points to avoid circadian clock regulation of gene expression.

### RNA-seq library preparation, Illumina sequencing, and *de novo* assembly

Total RNA extracted from crown buds using the pine tree extraction protocol [79] was used to prepare RNA-seq libraries for Illumina Next-Generation Sequencing. Total RNA was also used to prepare cDNA template through reverse transcription according to manufacturer’s instructions (Invitrogen). For library preparation, poly A^+^ RNA was isolated, reverse transcribed, and appropriate linkers were attached for Illumina sequencing using the NEBNext Ultra Directional RNA Library Prep Kit for Illumina (New England Biolabs Inc. Ipswich MA) according to manufacturer’s instructions with unique primers for each of the 15 samples. The resulting samples were pooled and 100 base paired end reads were generated on a single lane of Illumina by the Roy J. Carver Biotechnology Center, University of IL (http://www.biotec.illinois.edu/htdna). Initial read quality was assessed using the FastQC program (http://www.bioinformatics.babraham.ac.uk/projects/fastqc/) in the iPlant discovery environment [80]. The program Sickle-Quality-Base-Trimming [81] was used to trim reads for quality and length using the parameters of a minimum quality score of 20 and a minimum read length of 70 bases in the iPlant discover environment. Number of raw fragments and trimmed fragments are provided in Additional file 1: Table S1. To ensure that most complete transcriptome was assembled for use as a reference database (http://www.ncbi.nlm.nih.gov/geo/download/?acc=GSE71317&format=file&file=GSE71317%5FTrinity%5Fall%5FRNAseq%2Efasta%2Egz), trimmed reads from the above samples along with samples from several other RNA-seq studies on leafy spurge were combined into two files (one for each paired end) using the Concatenate Multiple Files program and the reads were kmer normalized using the program Trinity Normalize By K-mer Coverage [82] in the iPlant discovery environment with the default parameters of no more than 30 times coverage for a given kmer. The program Trinity [83] was then used to assemble the resulting paired end read files. This combined assembly was annotated by BlastX [84] against the nonredundant database with a minimum E value cut off of 10E-5. BlastX against the TAIR10 protein sequence database was also used to identify the most similar *Arabidopsis* genes with a similar E value cutoff. This assembly was used to map fragments and quantify sequences using the RSEM program suite [85] and the embedded program suite EBseq [86] was used to identify the probability that any given sequence was differentially-expressed between any sample groupings (see Additional file 8: Running RSEM for scripts displaying options used for expression analysis programs). The annotated assembled transcriptome and expression data (in transcripts per million) with differentially expressed genes noted by the posterior probability of the false discovery rate is provided in Additional file 2: RNA-seq master file.

For this manuscript, only component-based gene expression analyses were considered; however, contig-level expression analyses were also performed (data not shown, but are available through the Gene Expression Omnibus; see accession information below). For all subsequent analyses, only components with more than 10 hits per million in all samples from at least one time point were considered as expressed. It should be noted that there are a large number of components that do not represent open reading frames. Also, a small portion (~7 %) may have come from non-leafy spurge RNAs sources. However, these non-plant genes and non-coding transcripts were largely ignored by the required minimum expression level. To create a heat map for RNA-seq analysis, ratios of log2 transformed relative expression values for each treatment were used to compare to 0 h treatment. Heat-maps of the RNA-seq results were created using Eisen Lab software, Cluster and TreeView as described by Eisen et al. [87].

### RNA-seq data analysis

GeneMaths XT 2.1 software (Applied Maths Inc., Austin, TX) was used for principal component analysis of the normalized and trimmed dataset derived from the EBseq output. Pathway Studio software (http://www.ariadnegenomics.com) and AGI designations for *Arabidopsis* genes were used for Gene Set Enrichment Analysis (GSEA) of AraCyc pathways [88, 89] and for Sub-Network Enrichment Analysis (SNEA) [90]. GSEA is a statistical method to determine if predefined sets of genes are over-represented between treatments. The AraCyc component of GSEA is an *Arabidopsis* database that houses a large set of experimentally-supported and computationally-predicted metabolic pathways [88] (http://pmn.plantcyc.org/ARA/class-instances?object=Pathways). SNEA generates regulatory and interacting network relationships that facilitate interpretation of experimental data and development of new hypotheses [90] (http://www.ariadnegenomics.com/products/pathway-studio/expression-analysis/algorithms/). SNEA identify expression targets and small molecules over-represented in the above comparison datasets. We applied SNEA based on published results for *Arabidopsis*.

### Real-time quantitative PCR (RT-qPCR)

Gene expression by RT-qPCR and RNA-seq analyses were examined using total RNA prepared from UABs as previously described above. Leafy spurge homologs of *Arabidopsis* genes involved in hormone, growth, light, and temperature response/regulation were selected for analysis. Primer pairs (20–24 nucleotides) were designed using Lasergene (DNASTAR, Inc., Madison, WI) sequence analysis software from clones annotated to genes (Additional file 9: Table S7) based on sequences obtained from a leafy spurge EST-database [37]. The details of cDNA preparation and RT-qPCR parameters were described previously by Chao [91]. Briefly, the comparative CT method was used to determine changes in target gene expression in test samples relative to a control sample. Fold difference in gene expression of test vs. control sample is 2^-ΔΔCT^. SYBR green chemistry was used to produce fluorescent signal, and three technical replicates were used per sample for the RT-qPCR experiments. The C_T_ value of each gene is the average of three technical replicates. A previously verified leafy spurge SAND gene was used as an internal reference [92]. The difference in transcript abundance is designated as log2. Heat-maps of the RT-qPCR results were created based on log2 values using Eisen Lab software, Cluster and TreeView as described by Eisen et al. [87].

### Hormone measurement

Hormone measurements were performed by National Research Council of Canada (Saskatoon, SK) on a UPLC/ESI-MS/MS utilizing a Waters ACQUITY UPLC system. The procedure for quantification of multiple hormones and metabolites were described by Chiwocha et al. [93, 94]. Statistical analysis was done with PC-SAS using the ANOVA procedure. Means were compared with Tukey’s multiple comparison procedure or Dunnett’s t tests at *P* = 0.1 [95]. There were four biological replications per time point.

## Availability of supporting data

All supporting data are included as additional files. Raw and assembled RNA-seq data are available from the Gene Expression Omnibus under the accession number GSE71317 (http://www.ncbi.nlm.nih.gov/geo/query/acc.cgi?acc=GSE71317).

## Additional files

Additional file 1: Table S1. Number of raw fragments, number of trimmed fragments, and contig represented in each of the 15 RNA-seq samples. (XLSX 10 kb) Additional file 2:
**RNA-seq master file.** (XLSX 136875 kb) Additional file 3: Table S2. Heat map diagram showing changes in transcript abundance obtained by RNA-seq vs. RT-qPCR analysis. (XLSX 37 kb) Additional file 4: Table S3. Hormone-related genes that are significant and differentially-expressed based on RNA-seq analysis. (XLSX 245 kb) Additional file 5: Table S4. AraCyc pathways that are over-represented for comparisons 6 h vs. 0 h, 24 h vs. 0 h, and 72 h vs. 0 h based on GSEA. (XLSX 91 kb) Additional file 6: Table S5. Central hubs for expression targets and small molecules based on SNEA. (XLSX 24 kb) Additional file 7: Table S6. Differentially-expressed sugar transporter genes. (XLSX 18 kb) Additional file 8:
**Running RSEM.** (TXT 4 kb) Additional file 9: Table S7. Primers used for RT-qPCR analysis. (XLSX 38 kb)
